# Applicability of electro-osmotic flow for the analysis of the surface zeta potential†

**DOI:** 10.1039/c9ra10414c

**Published:** 2020-02-13

**Authors:** Olivija Plohl, Lidija Fras Zemljič, Sanja Potrč, Thomas Luxbacher

**Affiliations:** University of Maribor, Faculty of Mechanical Engineering, Laboratory for Characterization and Processing of Polymers Smetanova 17 2000 Maribor Slovenia olivija.plohl@um.si; University of Maribor, Faculty of Chemistry and Chemical Engineering Smetanova 17 2000 Maribor Slovenia; Anton-Paar GmbH Anton-Paar-Str. 20 A-8054 Graz Austria

## Abstract

The analysis of the surface zeta potential (SZP) opens up new possibilities in the characterization of various materials used for scientific or industrial applications. It provides at the same time insight into the material surface chemistry and elucidates the interactions with charged species in the aqueous test solution. For this purpose, an accurate, reliable and repeatable analysis of the SZP is the key factor. This work focuses on a detailed and systematic comparison of two electrokinetic techniques, *i.e.* the mapping of the electro-osmotic flow (EOF) and the measurement of the streaming potential (SP), for the surface zeta potential (SZP) determination of several materials with varying properties. Both techniques have advantages as well as drawbacks. The applicability of latex polymer material and inorganic tracer particles at varying ionic strength, the interaction between oppositely charged tracer particles and solid surfaces, the assessment of the pH dependence of the SZP and the isoelectric point (IEP), and the effects of sample porosity and conductance have been investigated. Although in some cases the EOF method gives a SZP similar to the streaming potential measurement, especially when the tracer particle exhibits the same charge as the solid surface, it was revealed that reliable results were only obtained with the streaming potential and streaming current method. Several obstacles such as elevated conductivity at higher ionic strength, the applied voltage for the EM measurement, and the nature of tracer particles lower the accuracy and reliability of the SZP determined by the EOF method. It was shown that the EOF method is not applicable to oppositely charged surface and tracer particles and also limited to low salinity conditions especially when using polymeric tracer particles. Although the EOF method does not require the formation of a capillary flow channel, it disables a non-destructive SZP of fragile or valuable samples, such as QCM-D sensors, in comparison to the SP approach.

## Abstract 2

Detail comparison of two different electrokinetic phenomena EOF and SP method for the SZP determination with taking into account various materials with different surface and bulk properties.

## Graphical abstract

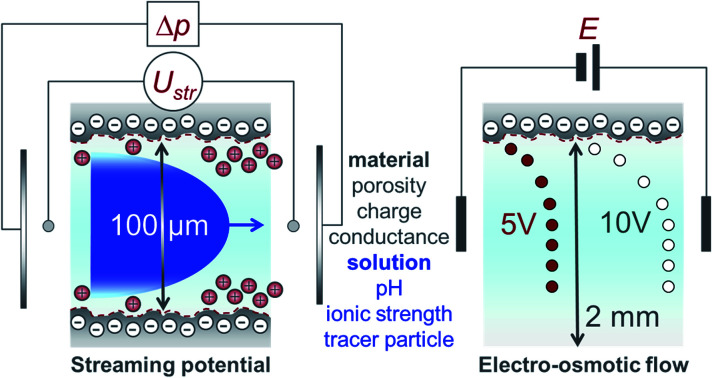

## 1. Introduction

When materials are brought into contact with an aqueous solution, they acquire a surface electric charge by different processes such as ionization, ion adsorption or ion dissolution. The need for charge compensation leads to the formation of an interfacial charge distribution in the aqueous phase that is described by the model of the electric double layer. Electrokinetic phenomena are induced by the movement of one of the phases (solid or liquid) relative to the second phase. The electrokinetic behaviour depends on the electric potential at the shear plane between the charged surface and the electrolyte solution. This potential at the shear plane is called the electrokinetic or *ζ* potential (ZP).^1^ Different electrokinetic effects exist depending on the way how the movement is induced. Electrophoresis, electro-osmosis, the streaming potential (SP) and electroacoustic represent the four electrokinetic measurement techniques from which zeta potential (ZP) is derived.^1,2^

In general, ZP represents the charge at the solid–water interface that affects materials functionality and at the same time is the crucial parameter for the determination of the material's isoelectric point. Surface zeta potential (SZP) analysis is a vital method for qualifying important features of new materials in technical (*e.g.*, effects of fouling and cleaning of membranes used for water treatment,^3,4^ textile industry^5–7^) and biomedical applications (*e.g.* biofilm formation, haemocompatible implants^8–11^). Furthermore, it enables to gain insights into modification processes that result from surface treatment or surface interactions with biological or natural environments under near-ambient conditions.^1,2^ Thus, SZP is a key parameter for understanding surface properties and for developing new specialized materials, *e.g.*, biomaterials that get in contact with blood, or virus retention filters in biopharmaceutical segments that specifically require accurate, reliable and reproducible macroscopic surface SZP analyses.

Materials macroscopic SZP is commonly determined using the established streaming potential technique. The measurement of the SP (and alternatively of the streaming current) is the direct approach to the SZP, where liquid flow through a capillary generates an electric potential. A pressure gradient is applied between both ends of a capillary flow channel, which generates liquid flow and the streaming potential signal. This electrokinetic effect is used to assess the surface charge of macroscopic solids with a flat surface, but also with more complex surfaces such as porous material, fibers, and granular media.^12^ Recently, an alternative indirect measurement technique for SZP of flat surfaces was introduced,^13^ and is comprising phase analysis light scattering (PALS) using mostly polymeric tracer particles through electro-osmotic flow (EOF) mapping in a simple dip cell arrangement. Several studies were reported on using EOF for SZP determination in a dip cell,^13–21^ in a coated microchannel that measures the mobility of known ZP of tracer particles close to the surface,^22^ in a quartz cell having mostly non-ionic hydroxypropyl-coated polystyrene tracer particles^23–35^ or with microelectrophoresis instrumentation modified to accommodate quartz capillaries using sulphated polystyrene latex particles.^36^ It relies on the principle that the electrophoretic mobility of tracer particles dispersed in a liquid is affected by the surface when these particles approach the solid sample. Each technique shows advantages and drawbacks. The EOF method requires an apparently smaller sample size compared to the SP method but disables a non-destructive zeta potential analysis of fragile or valuable samples, *e.g.*, QCM-D sensors.^37^ The solid sample is held in such way that if tracer particles sediment they do not deposit on the sample surface.^13–15^ The SP method is very competitive due to its versatility to determine the surface zeta potential for various geometries of solid samples such as flat surfaces, fibers, or granular media. Despite of the known reputation of the SP method, there the following drawbacks are proposed in the literature when using this method. These were correlated to the needs for a careful sealing of the sample in the measuring cell to accept the applied pressure gradient, to the limited sensitivity at higher salinity of the aqueous test solution, and that the SP technique typically requires an instrument that is solely dedicated to the SZP analysis.^13,14^ However, for a state-of-the-art SP analyser, these drawbacks have been overcome. On the other hand several authors have reported SZP results for various material types (^13–21,23–35^) obtained by the EOF method (in different configurations) using mostly polymeric tracer particles. The problems observed in these studies were associated with a high measurement uncertainty that consequently lowers the quality of the data^15,16,24,27,38–40^ especially for more complex sample surfaces (nanofibers, membranes), difficulties in determining the surface IEP when oppositely charged tracer particles were exposed,^15,18,19,26,29^ or the observation of polymeric tracer particles sedimentation and degradation (colour change of tracer particle dispersion), which is even more pronounced under elevated ionic strength,^15,19,24,28^ that limits the applicability of the EOF method at high salinity conditions. Moreover, results from the EOF method showed also bad reproducibility and questionable reliability.^17,28–31,33^ Results were of varying quality, for instance with 14% difference in the SZP when using two different tracer particles under otherwise same measurement conditions in the dip cell^19^ and frequently compared with results obtained by the SP method, just to mention a few.^13–21^ Additionally, research showed that EOF is not applicable to oppositely charged surface and tracer particles.^22^ In another paper the need for the selection of non-ionic tracer particles was also pointed out in order to avoid electrostatic adsorption.^20^ As an example it was not possible to observe a positive zeta potential below the IEP of glass (negatively charged at neutral pH) using negatively charged tracer particles.^26,34^ Taking all these limitations into account, the necessity for a reliable, repeatable and reproducible SZP with minor measurement uncertainty is of particular concern, especially for the characterization of surface properties of more delicate materials such as membranes or biomaterials.

In spite of the already available investigations that compared the SP method with the EOF technique in the dip cell arrangement with few types of materials, such as different polymeric membranes,^15^ glass^13,14^ and PVDF foil,^13^ a comparison between both techniques for a wider range of materials and the study of the individual influences on the measurements is still missing. To our knowledge, no such study on the detailed comparison of the EOF and the SP methods for a range of inert, conductive and highly porous materials with diverse properties has been undertaken yet.

The comparison of both electrokinetic techniques, EOF and SP, was conducted on various materials (*i.e.* polyamide thin-film composite membrane, pristine and chitosan-coated polypropylene foil, cellulose acetate filter, silicon oxide wafer and Ni-based alloy) that cover a wide range of possible applications (microfilters for biopharmaceutical applications, membranes for desalination, food packaging, wet processing of semiconductor wafers, *etc*) and challenge both measurement techniques. In this way, the role of the chemical nature, the size and the charge of tracer particles as a function of ionic strength, pH, solid material's IEP, porosity and conductivity was examined. The SZP obtained with the EOF method was compared to the SZP determined from the SP measurement. For all measuring conditions we focused on the reliability, reproducibility and accuracy of the obtained SZP from both measuring techniques.

## 2. Materials and methods

FeSO_4_·7H_2_O was purchased from Riedel-De Haen, Fe_2_(SO_4_)_3_·7H_2_O, HCl (≥37%), tetraethylorthosilicate (TEOS, ≥98%) were all purchased from Sigma-Aldrich. NH_4_OH (25% aqueous solution), NaOH (>98%) and acetone (≥99.5%) were purchased from Honeywell. Absolute EtOH (anhydrous) was obtained from CarloErba and citric acid (≥99.5%, water free), KCl, HCl, and KOH were purchased from Roth. Albumin V from bovine serum (BSA) was purchased from Merck KgaA (USA). All chemicals were used as received, without any further purification. Ultrapure water (with a resistivity of 18.2 MΩ cm obtained from Milli-Q, Millipore Corporation, Massachusetts, USA) was used throughout the experiments. A latex dispersion standard with a conductivity of 0.4 mS cm^−1^ and pH 9 was provided by Anton Paar GmbH. A silica nanoparticle dispersion was prepared from TM-40 colloidal silica, 40 wt% suspension in H_2_O (Ludox, Sigma-Aldrich), with a conductivity of 4.85 mS cm^−1^ and pH 8.5–9.5.

### 2.1 Solid samples

One of the initial goals to use the SZP analysis was the characterization of the surface and interfacial charge of flat sheet polymer membranes.^3^ We therefore included a polyamide thin-film composite membrane for reverse osmosis (SW30-HR, Dow Chemical, USA). For equilibration the RO membrane was soaked in Milli-Q water prior to the SZP analysis. A polypropylene film with 50 μm thickness was obtained from Goodfellow (Huntingdon, UK). A single-side polished silicon wafer (150 mm, thickness 675 μm) with a 1000 Å thick silicon oxide coating was cut into pieces of 20 mm × 10 mm (Siegert Wafer, Aachen, Germany). Cellulose acetate microfiltration membranes with 0.2 μm and 0.45 μm pore size were obtained from Sartorius (Göttingen, Germany). A foil of Hastelloy C-276 (NiMoCr alloy, thickness 1 mm) was purchased from GoodFellow, UK.

### 2.2 Preparation of chitosan-based PP foil

To achieve positive charge of the surface, the PP foil was functionalized with coating based on polysaccharide chitosan. The surface of PP was first cleaned ultrasonically in a bath of 70% ethanolic solution for 5 min, afterwards dried in an oven at 40 °C and finally activated for 20 min using ultraviolet-ozone surface treatment; during the activation process, the surface becomes more hydrophilic and consequently better adhesion of dispersions is obtained. The PP foil was functionalized in two layers (layer-by-layer composition): as a (1) layer 2 wt% chitosan macromolecular solution was applied and (2) layer was formed with dispersion of chitosan nanoparticles with embedded cinnamon extract. All the details of a solution preparation are presented in our recently published paper.^41^ After application of each layer the foil was dried at room temperature. For modification of the surface, the method of printing using a magnet was used (roll printing).

### 2.3 Preparation of tracer particle dispersion

#### 2.3.1 Latex tracer particles

The conductivity of the latex tracer particle dispersion was 0.4 mS cm^−1^, which corresponds to an aqueous KCl solution with an ionic strength of 2 mM. The latex tracer particles were diluted volumetrically by the same amount of predefined KCl solution, in order to obtain an ionic strength 1, 2, 5 and 10 mM. Specifically, the 0.5 mL of the tracer particles was diluted by 0.5 mL of selected KCl solution to achieve the appropriate final ionic strength. It has to be pointed out that the exact mass content of the latex particles was unknown but remained constant in the series of particle dispersions of different ionic strength. For each SZP measurement, the solid sample was rinsed several times with Milli-Q water and a fresh dispersion of the tracer particles was prepared.

#### 2.3.2 1 wt% Ludox

The 1 wt% Ludox tracer particle dispersion was prepared by diluting the 40 wt% stock solution with 2 mM KCl. The pH of the dispersion was adjusted with 0.05 M KOH or 0.05 M HCl to pH 3, 5, 7 and 9.

#### 2.3.3 0.1 wt% MNPs@SiO_2_

Magnetic nanoparticles based on maghemite (MNPs) were synthesized under air atmosphere by the coprecipitation of Fe^2+^ and Fe^3+^ ions. Briefly, an aqueous solution of Fe^2+^ and Fe^3+^ (*V* = 250 mL) with a molar ratio of *n*(Fe^2+^) : *n*(Fe^3+^) = 2.4 : 1 ratio was prepared by dissolving iron sulphate in ultrapure water. Afterwards, diluted aqueous ammonia solution was slowly added to iron salts solution at pH 3 to precipitate iron hydroxides. For the formation of MNPs, 125 mL of ammonia solution (25%) was added to the above mixture and additionally agitated with a magnetic stirrer for 30 min. As-prepared MNPs were cleaned several times with diluted ammonia solution and ultrapure water. Then, the stable colloidal dispersion of MNPs was prepared using adsorption of citric acid. Here, 0.6 g of as-prepared bare MNPs were redispersed in 30 mL of ultrapure water and 2.5 mL (0.5 g mL^−1^) citric acid aqueous solution was added. The pH was raised to 5.2 with diluted ammonia solution and put under reflux for 1.5 h at 80 °C. After refluxing, the pH of cooled dispersion was set to ∼10 with ammonia solution (25%). Stable MNPs were then coated with thin silica shell (MNPs@SiO_2_). NH_4_OH was added to stable MNPs dispersion (15 mg mL^−1^, pH = 10.6). The mixture was agitated for 15 min and added rapidly to the solution of EtOH and TEOS (10 mg mL^−1^). This was followed with pH setting to 10.6, using 25% NH_4_OH. The coating process was left to proceed for 2 h under continuous stirring. The obtained core–shell MNPs@SiO_2_ were cleaned to remove the excess reagents using absolute EtOH and ultrapure water. The details about the preparation of the MNPs@SiO_2_ and about their characteristics can be found in the following ref. 42 and 43. For the EOF analysis, 0.1 wt% dispersions of MNPs@SiO_2_ in 2 mM KCl were prepared with the pH adjusted to pH 3, 5, 7 and 9. Prior to be used as a tracer particle dispersion and to remove possible agglomerates, the dispersion was additionally filtered using a 1 μm filter.

#### 2.3.4 1 wt% BSA in 2 mM KCl

A 1 wt% BSA solution in 2 mM KCl was prepared from Albumin Fraction V powder. pH 3, 7 and 9 of the BSA solution was adjusted with 0.05 M KOH or 0.05 M HCl. Prior to use, the BSA solution was filtered through a 0.25 μm membrane filter to remove any possible present agglomerates.

### 2.4 Electro-osmotic flow mapping

The EOF experiments were performed with a Zetasizer Nano ZS equipped with a He–Ne laser (*λ* = 633 nm) and the SZP accessories (Malvern, UK). The signal for dynamic light scattering (DLS) was detected at 173° and for the electrophoretic mobility measurement at 13°. The SZP analysis were conducted by the method described by the Corbett *et al.*^13^ Briefly, for the SZP analysis the sample was cut into rectangular pieces not larger than 7 mm × 4 mm (L × W) and attached *via* a double-sided adhesive tape to the sample holder (7 mm × 4 mm, poly(ether ether ketone), PEEK) perpendicular between the electrodes of the dip cell. The completed SZP cell was then placed into the electrophoretic light scattering (ELS) instrument. It should be noted that special care was taken not to damage the sample surface during the attachment to the sample holder. A coarse alignment of the zero position of the sample stage was performed using the height alignment tool. A disposable cuvette was filled with 1.2 mL of the prepared aqueous tracer particle dispersion and then the SZP cell was inserted. The latter should be done by tilting the cuvette in order to avoid any bubbles being caught between sample and electrodes, and to ensure that the sample plate was entirely submerged. The final alignment of the sample stage was performed using the count rate meter of the Zetasizer software (version 7.12). It should be noted that special attention has to be paid for the fine alignment since hitting the sample surface with the laser beam gives a false indication about the zero-position location that significantly lowers the quality of the data. The attenuator was set to 11 (*i.e.*, the full intensity of the laser beam was used) and forward scatter was selected for monitoring the ELS signal. The number of ELS runs for the EM measurement was selected automatically and the measurements of the EM for each surface displacement was repeated 3 times. The SZP was evaluated from the measurement of the EM at 4 displacements with a step size of 125 μm. For the EM measurement the applied voltage, the attenuation of the laser beam, and the number of consecutive runs were adjusted automatically with 3 repetitions at 1500 μm displacement. The apparent EM of the tracer particles (which may be converted to an apparent zeta potential) was measured at surface displacements of 125, 250, 375 and 500 μm using the slow field reversal mode while the tracer particle mobility itself was measured at 1500 μm using the fast field reversal mode. The measurement relies on the assumption that the EOF at the solid surface decays with increasing distance from the surface. Close to the surface the velocity of the tracer particles will be dominated by the electroosmotic flow, while at distances far away from the surface it will be dominated by its EM.

The apparent – zeta potential *ζ*_apparent_ is calculated at each displacement from the mobility measurement by

1 *ζ*_apparent_ = *μ*_apparent_*η*/(*ε*_rel_*ε*_0_)

where *μ*_apparent_ is the apparent EM, *η* is the solution viscosity, *ε*_rel_ is the dielectric coefficient of the solvent, and *ε*_0_ is the vacuum permittivity.

The SZP is then calculated by eqn (2) using the zeta potential of the tracer particle (determined at a distance far from the surface) and the intercept on the *y*-axis, which is obtained by a linear extrapolation of the experimental data for the particle mobility at various distances from the solid surface.

2 Surface zeta potential = −intercept + tracer zeta potential

### 2.5 SP and streaming current method

The measurement of the SP *U*_str_ (and alternatively of the streaming current *I*_str_) is the direct approach to the SZP. Liquid flow through a capillary generates an electric potential. This electrokinetic effect is used to assess the SZP for macroscopic solids with a flat surface. A pressure gradient Δ*p* is applied between both ends of a rectangular flow channel, which generates liquid flow and the SP signal. The SP is recorded within a range of pressure differences and the slope of the linear dependence (the SP coupling coefficient d*U*_str_/dΔ*p*) is used to calculate the SZP according to eqn (3):

3 *ς* = d*U*_str_/dΔ*p* × *η*/(*ε*_rel_ × *ε*_0_)*κ*_B_

*κ*_B_ is the electric conductivity of the aqueous test solution.

The SP measurements were performed with SurPASS 3 (Anton Paar GmbH, Austria) using the adjustable gap cell for mounting samples with a flat surface. A pair of each sample with a size of 20 mm × 10 mm was mounted on the sample holder using double-sided adhesive tape. The distance between sample surfaces was adjusted to 110 ± 10 μm. The electrolyte solutions were 2 and 10 mM KCl and the pH was automatically adjusted with 0.05 M KOH and 0.05 M HCl. Prior to the measurement the solid sample was equilibrated at neutral pH with several rinsing steps and then pH was set to the alkaline range. A pressure gradient of 200–600 mbar was applied to generate the SP or the streaming current, which was measured using a pair of AgCl electrodes. For each individual pH, 3 measurements were performed and the average SZP is reported. The electrolyte pH and conductivity were monitored using pH and conductivity probe, and all the experiments were done at room temperature. Between individual sample analyses, the electrolyte system was thoroughly rinsed with ultra-pure water to ensure that any prior solution was removed.

## 3. Results and discussion

### 3.1 Verification of the EOF method

#### 3.1.1 Latex tracer particles properties

Polymer latex dispersions are commercially available and frequently used as tracer particles for EOF mapping of the SZP.^13–21^ The hydrodynamic diameter of the latex tracer particles as function of ionic strength did not differ significantly and was found to be around 350–360 nm (Fig. SI1a and b†). It can be seen that the latex particles exhibit a narrow size distribution with no agglomerates present. Additionally, the ZP of the latex tracer particles was shown to be around −45 mV in 2 mM KCl at pH 9 and similar magnitude was observed regardless of the ionic strength (Fig. SI1a and c†). Since the hydrodynamic diameter but also the ZP of the polymer latex particles did not change significantly within the selected range of ionic strength (1–10 mM), these probe particles showed suitable characteristics for being used as tracer particles for EOF mapping.

#### 3.1.2 Reverse osmosis (RO) membrane

**Fig. 1:**
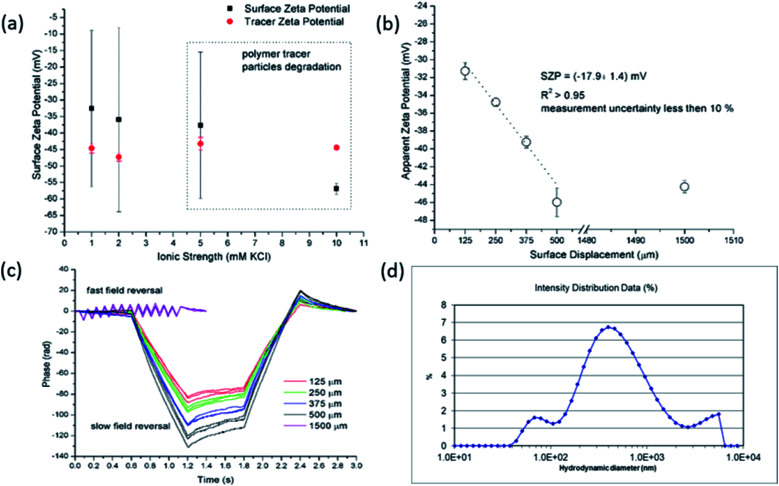
SZP results from EOF measurements for RO membrane using latex tracer particles as a function of the ionic strength (a). Exemplarity apparent zeta potential at different displacement in 2 mM KCl (b) and corresponding phase plots (c). Size distribution of the latex tracer particles after EOF measurement at 5 mM ionic strength (d).

We have exemplarily selected a commercial thin-film composite flat sheet polyamide membrane for reverse osmosis as a solid sample for studying the effect of the ionic strength as this knowledge is of paramount importance for the application of the RO membrane at environmentally relevant conditions. For this purpose, the skin side of the RO membrane was subjected to the EOF analysis (Fig. 1a). Here, the parameters of the EOF mode of measurement were set to 4 surface displacements (125, 250, 375, 500 μm) while the EM of the tracer particles was measured at 1500 μm (far away from the membrane surface). The applied voltage was automatically set to 10 V by the software. Fig. 1a shows the SZP for the skin side of the RO membrane using latex tracer particles at different ionic strength. Each measurement at the defined ionic strength was performed 3 times, always using a freshly prepared tracer particle dispersion. An example of the measurement in 2 mM KCl that meets the quality criteria for EOF mapping is shown in Fig. 1b and c. The correlation coefficient of the linear regression fit exceeds 0.95 with a measurement uncertainty less than 10% (Fig. 1b), and the negative phase plots are well defined with minimum noise (Fig. 1c). Nevertheless the reproducibility of the SZP in 2 mM KCl at pH 8.5 was found much worse (*ζ* = −37 ± 30 mV). For comparison, the SZP analysis of the RO membrane with the SP measurement in 2 mM KCl resulted in *ζ* = −25 ± 0.1 mV at pH = 8.5. Indeed as a rule of thumb the stability of common particle dispersions is limited if the magnitude in the zeta potential assumes <25 mV. This physical phenomenon is thus among the main constraints that restrict the applicability of EOF mapping to be used in a wide range of measuring conditions determined by pH and ionic strength. Surprisingly, after using the latex particles in 5 mM KCl for the SZP analysis by the EOF method in the dip cell arrangement we observed the colouring of the latex dispersion (Fig. SI2†). The same trend was also observed at an ionic strength of 10 mM with an even higher degradation degree. Moreover a broadening in the size distribution of the tracer particles after measurement was clearly revealed by the DLS measurement (Fig. 1d). From these results we assume that high local temperature (Joule heating) and a concentration of particles at the electrode occur during the EOF measurement, which consequently lead to the polymer particle degradation. This is consistent with the observation by Thomas *et al.*^15^ at elevated ionic strengths using polymeric tracer particles. In fact, Vasconcelos *et al.*^19^ found that the conductivity for a reliable SZP using polymer tracer particles should be in the range of 200–300 μS cm^−1^ (which corresponds to 2 mM KCl), which supports our observation of the loss of integrity of the polymer latex tracer particles. The degradation of tracer particles likely adds to the reasons that contribute to the large measurement uncertainty although the latter is already pronounced at lower ionic strength. On the other hand, the ZP of the tracer particles (measured during the EOF mapping) remained almost constant at *ζ* = −45 mV at elevated ionic strength (Fig. 1a). First we did not exclude that changes in the polymer tracer particle integrity may be caused by the RO membrane sample. Therefore we continued testing the flat sample holder of the dip cell made of PEEK for the EOF measurement using the same conditions as in the case of the RO membrane. At 5 mM KCl, we again observed a colouring of the tracer particle dispersion and the occurrence of brownish agglomerates after the EOF measurement (Fig. SI4a†). These results indeed indicate that the tracer particle degradation was not caused by the solid sample but occurred most likely due to the increased ionic strength. In the Uzgiris dip cell arrangement^44^ the applied voltage determines the strength of the electric field between the electrodes and therefore the EM of the tracer particles. The SZP analysis in 5 mM KCl was performed with a voltage of 10 V (this was automatically set in the Zetasizer software). To see the effect of the applied voltage on the stability of the polymer latex tracer particles, we manually adjusted the voltage to 5 V, 10 V and 20 V, thereby keeping the ionic strength at 5 mM. The results of these experiments shown in Fig. SI4b† revealed an even worse behaviour at a higher applied voltage with a completely different size distribution of the latex tracer particles in 5 mM KCl after completing the measurement (Fig. SI5†). The latter clearly indicates the unsuitability of this polymeric standard being used as tracer particles due to its degradation. The first change of the size distribution of tracer particles was observed already at 5 V, while by increasing the voltage, the size distribution starts to broaden indicating the presence of agglomerates (Fig. SI3†). Moreover, with higher applied voltage also the linear regression fit resulted in a lower value of the correlation coefficient, and the measurement uncertainty started to increase (Fig. SI4b†).

Preliminary experiments clearly revealed that the standard polymeric latex tracer particles are not suitable to be used as tracer particles for the determination of the SZP using the EOF mode under certain conditions – especially in media close to ambient conditions, where the materials' surfaces are exposed to even more complex aqueous environments. However, this is of paramount importance for measurements that allow for the phenomenological assessment of the solid materials' properties that are mainly used for industrial purposes. Here, the accurate and reliable SZP analysis is important for the prediction of, *e.g.*, membrane performance. The lack of proper measurement can be in the first place attributed to polymeric tracer particle degradation (the degradation of the electrodes that are integrated in the dip cell was excluded) that significantly influences the absolute value and interpretation of the SZP. Further research was therefore focused on finding more inert, stable and appropriate inorganic tracer particles with a suitable size and a negative charge. It should be also pointed out that further experiments were limited to 2 mM ionic strength in order to achieve reliable data with EOF mapping.

### 3.2 Comparison of EOF and SP: influence of different “novel” effects

#### 3.2.1 Characterization and effect of inorganic and protein tracer particle dispersions

**Fig. 2:**
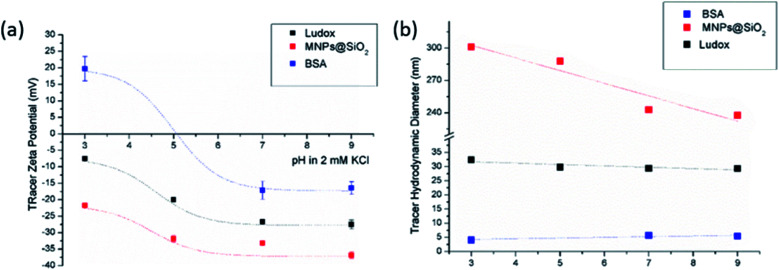
Tracer ZP (a) and hydrodynamic diameter (b) as a pH function at initial 2 mM KCl electrolyte solution.

Due to the unsuitability of polymer tracer particles, the aim was to find inorganic and stable monitoring particles for the EOF method. Fig. 2 shows the ZP and hydrodynamic diameter of Ludox, MNPs@SiO_2_ and BSA tracer particles at different pH values, ranging from acidic to alkaline. The ZP of both silica-based NPs (Ludox and MNPs@SiO_2_) exhibit similar trends with MNPs@SiO_2_ being more negative when compared to Ludox tracer particles (colloidal silica). For both NPs the IEP is expected to be around pH 2.^42,43^ In this way the aforementioned tracer particles possess negative charge in the studied pH range due to the acidic silanol groups of silica and the silica coating. Due to the fact that also proteins can be used to indirectly monitor the SZP, the amphoteric BSA was selected as a protein with its IEP at pH 5. Below pH 5, BSA is positively charged while above its IEP it is negatively charged. In spite of the decreasing magnitude of the ZP at lower pH, the hydrodynamic diameter of BSA as well as of the Ludox tracer particles did not significantly change but remained stable across the complete pH range. On the other hand MNPs@SiO_2_ showed an increasing hydrodynamic diameter when lowering pH as a consequence of the lower repulsive forces among particles that leads to agglomeration. Ludox tracer particles exhibit a hydrodynamic diameter of around 30 nm, BSA around 4 nm, while the hydrodynamic diameter of MNPs@SiO_2_ increased from 240 to 300 nm with lowering pH. It has to be pointed out that all the particles were initially dispersed in 2 mM KCl electrolyte solution, however, the conductivity of the dispersion changed when adding base or acid to adjust the proper pH values. Regarding the mass fraction of the tracer particles in dispersion, 0.1 wt% for MNPs@SiO_2_ and 1 wt% for Ludox give high enough count rates so that sufficient diffracted light intensity was detected.

**Fig. 3:**
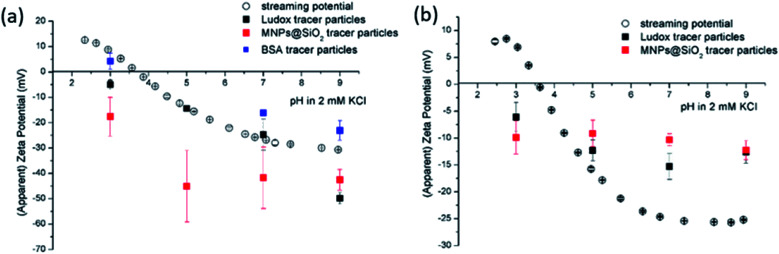
Surface zeta potential for (a) a polypropylene foil and (b) a reverse osmosis membrane determined from streaming potential measurement (empty circles) and EOF mapping (filled squares) using different tracer particles (black: Ludox, red: MNP@SiO_2_, blue: BSA). The symbols represent the average zeta potential of three measurements and the error bars the corresponding standard deviation. For the colour code the reader may refer to the electronic version.

The size effect of two different inorganic tracer particles (MNPs@SiO_2_ and Ludox) with similar surface chemistry together and of a protein (BSA) was studied on a polypropylene (PP) foil, which exhibits a simple, non-complex flat and inert polymer surface (Fig. 3a), and was suggested as a reference material for the non-destructive SZP analysis by the SP method.^45–47^ The SP data for the PP foil show minimum measurement uncertainty (less than 10%), good repeatability (each point is the mean value of three repetitions) and the IEP at pH 3.8, which is typical for polymers.^48^ Oppositely, larger deviations were observed for the indirect EOF method for all three types of tracer particles. A significantly larger measurement uncertainty was determined using the MNPs@SiO_2_ particles, which exhibit the largest particle size. Otherwise all three results follow a similar trend as seen in the SP measurement. For instance, at pH 7 the measurement uncertainty was 29% with magnetic particles, 24% with Ludox and 6% with BSA (Fig. 3a). In comparison the SZP determined from SP measurements shows a repeatability of 1.1%. Although smaller in size, BSA showed the smallest deviation in comparison to the SP measurements, however, problems were observed with the EOF method that were associated with the protein deposition onto the palladium electrodes of the dip cell. Ludox and magnetic tracer particles were therefore selected to study solid materials with a more complex surface behavior, such as the RO membrane (Fig. 3b). Similarly as in the case of the PP foil, SP data show reliable results with minor measurement uncertainty exhibiting an IEP and the magnitude of the SZP that are common for such kind of polyamide thin-film composite membranes.^49^ On the contrary, results provided from EOF mapping showed a significant deviation from the SP results, but with a smaller measurement uncertainty as in the case of the PP foil. For instance, at pH 7 a standard deviation of 16% was determined with Ludox tracer particles, and 11% with MNPs@SiO_2_. Both types of tracer particles showed negative phase plots at all surface displacements at pH > IEP similarly as represented in Fig. 1c for the latex tracer particles. These results indicate that also small tracer particles can be used opposite to the suggestion by Mateos *et al.*^14^ who concluded that smaller tracer particles with a small absolute value of the ZP are not applicable for the EOF method due to the high effect of the EOF onto tracer mobility. Besides this, one should also take into account the effect of ionic strength that contributes to the EM of tracer particles. Interestingly, regardless of the sample surface tested, with negatively charged tracer particles neither the IEP nor the oppositive sign of the SZP for positively charged surfaces was achieved (Fig. 3) and can be related to possible Ludox particles attachment to positively charged surface. Moreover, if we compare the ZP trend of the tracer particles (Fig. 2a) with the SZP determined by the EOF method, a similar behaviour can be seen. In the case of the RO membrane larger deviations in the SZP were observed for magnetic nanoparticles when compared to SP data, thus in continuation, Ludox was chosen as an optimal tracer particle, which provided more accurate data.

#### 3.2.2 Determination of solid sample IEP

**Fig. 4:**
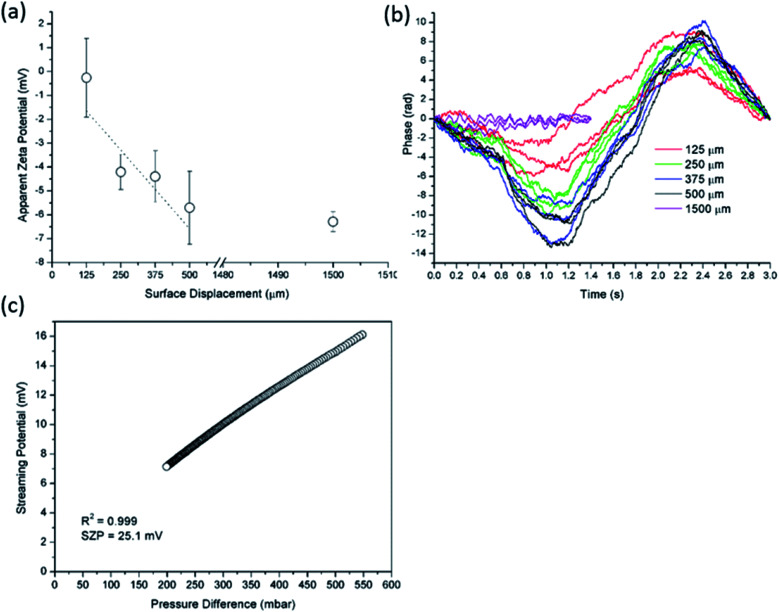
Apparent zeta potential *versus* surface displacement for EOF method using Ludox tracer particles at pH 3 and initial 2 mM ionic strength for PP foil (a). In (b) corresponding phase plots are illustrated. The pressure ramp at pH 3 for PP foil obtained from SP measurement is shown in (c).

For the selection of the tracer particles and the appropriate pH range for the SZP analysis we have to take into account the (initially unknown) IEP of the macroscopic solid surface and the surface charge behaviour of the tracer particles as a function of pH, as these properties are intertwined to each other. Ludox tracer particles show the IEP at pH ≈ 2 while the pH range for the SZP analysis was pH > 3. In other words, Ludox tracer particles are negatively charged in entire pH range studied. This allowed us to exclude the effect of the tracer particles' IEP. Generally, the determination of the IEP by the SP method presents no complications (Fig. 4). Oppositely, the IEP of various materials could not be achieved by the EOF method using either Ludox or MNPs@SiO_2_ as revealed from Fig. 3. This is exemplarily shown for the PP foil and the RO membrane. The same phenomenon was also observed for other studied materials, such as microfilters or the silicon wafer. Fig. 4a represents the linear fit of the apparent ZP at pH 3 for four surface displacements that allows indirectly for the calculation of the SZP by eqn (2) as *ζ* = −(6.78 ± 1.54) mV with the correlation coefficient of the linear fit lower than 0.95 (*R*^2^ = 0.823). The bad correlation is in agreement with the corresponding phase plots, which are undistinguishable and show a high degree of noise (Fig. 4b). Considering the IEP of the PP foil we expect a positive SZP as obtained by the SP measurement. Such discrepancy was already reported for polymeric membranes.^15^ Therefore, the results obtained by the EOF method significantly deviate from the expected SZP. The slope of the dependence of the SP on the differential pressure for the PP foil at pH 3 clearly indicates a positive SZP (Fig. 4c). The problematic determination of the IEP has also been pointed out by other authors who reported that it was impossible to achieve the IEP using the EOF method in a microchannel configuration with oppositely charged tracer particles and macroscopic surface.^22^ The IEP could only be determined by extrapolation to *ζ* = 0 mV.^19^ Another disadvantage of the EOF method is introduced by the increased conductivity at lower pH that causes changes in the inorganic tracer particles' integrity (colouring and agglomeration of Ludox and MNPs@SiO_2_ tracer particles) at pH 5 and 3. This degradation has a negative impact on the quality of the obtained data and requires to use a fresh inorganic tracer particle dispersion for each separate measurement. Interestingly, by comparing the magnitude of the ZP of the tracer particle (Fig. 2a) and the solid surface (Fig. 4a), the similarity can be clearly recognized. The latter can possibly indicate an adsorption of negatively charged Ludox tracer particles onto the positively charged PP foil at pH 3. Hiratsuka *et al.*^22^ reported on a progressive attachment of tracer particles onto an oppositely charged solid sample when using the EOF method in the microchannel configuration. As a next step we studied the effect of Ludox tracer particles on a positively charged solid surface at pH and ionic strength of the aqueous solution appropriate for the EOF method.

#### 3.2.3 Effect of opposite charge of solid surface

**Fig. 5:**
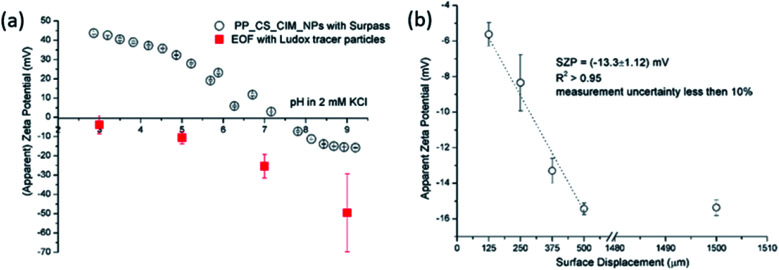
SZP of chitosan-based functional coating on PP foil determined with the SP and EOF methods using Ludox tracer particles (a). Exemplarily shown apparent zeta potential *versus* surface displacement for EOF measurement at pH 5 in 2 mM ionic strength using Ludox tracer particles (b).

The knowledge of an accurate SZP, for instance in active food packaging applications, where polymer films are accordingly modified to exhibit special features that prevents food spoilage^50^ is of paramount importance. The polymer films are most of the times modified with different functional coatings and successful surface modifications can be followed by the SZP. Chitosan is commonly applied as an antimicrobial coating.^51–53^ Therefore the SZP of a PP foil modified with chitosan, which introduces cationic charge (*i.e.* PP coated with chitosan NPs with embedded cinnamon extract) was determined in order to investigate the effect of an opposite charge between the macroscopic surface and tracer particles. The SP method allowed for a straight determination of the SZP with minor relative measurement uncertainty (less than 10% for three repetitions at each pH) and the IEP at pH 7 (Fig. 5a). This is in agreement with the expected behaviour of the chitosan-based coating on the PP foil and confirmed by the zeta potential analysis by ELS and the IEP of chitosan nanoparticles in dispersion.^54^ Very different SZP of the same highly positive chitosan-modified PP foil was determined by the EOF method with negatively charged Ludox tracer particles (Fig. 5a). Despite of lower error bars observed when approaching the acidic area, at all pH including the pH range where positive surface charge is expected the SZP maintained a negative sign. Even more, the linear regression fit and SZP data at pH 5 (Fig. 5b) were indicated as a good quality data according to EOF mapping with relative measurement uncertainty less the 10% (Fig. 5b) with well-defined negative phase plots (similar to Fig. 1c). In general, when two materials possessing different charge are brought into vicinity, the Coulomb attractive forces occur. From this effect it can be concluded that one has to select in advance proper tracer particles of the same sign of the charge and also has to know the behaviour of the solid surface. Otherwise, it is difficult to say which result is feasible or just an artefact if there is no direct comparison with the more conventional SP method.

#### 3.2.4 Effect of solid sample porosity

**Fig. 6:**
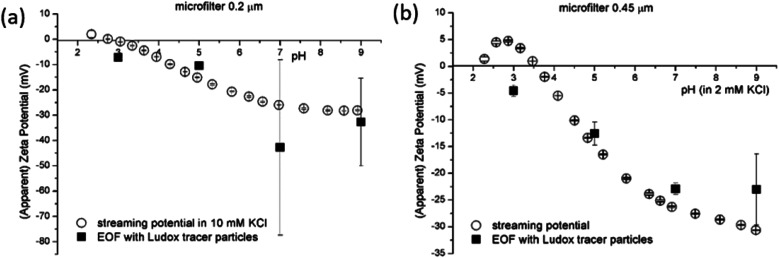
SP and EOF methods for SZP determination for microfilters with pore sizes 0.2 μm (a) and 0.45 μm (b).

The knowledge of effective SZP of porous surfaces is important for membrane processes as the latter has a significant influence on filtration processes. Here, bulk material porosity affect the SZP analysis where ionic conductance is introduced after exposure of the material surface to an aqueous solution.^55^ For this reason, microfiltration membranes with two different pore sizes were studied in terms of the SZP by both methods (Fig. 6). A cellulose acetate microfiltration membrane (0.2 μm pore size) and a microfilter with 0.45 μm pore size (Fig. 6) were explored to see the effect of solid sample porosity using both methods. For EOF the negatively charged Ludox tracer particles were used. When comparing the results of the SP and EOF methods, the SP measurement shows negligible error bars and an acceptable IEP, which is expected due to the filter composition.^56^ On the other hand, significantly larger relative uncertainties can be observed for the EOF method for both microfiltration filters especially in alkaline range. Although the general trend of the SZP obtained with the EOF method follows the SP behaviour, Fig. 6a shows large error bars at pH 7 that resulted in 81% relative uncertainty. This is a too large uncertainty in order to provide reliable data. For the microfilter with the nominal pore size of 0.45 μm a relative uncertainty of 29% at pH 9 indicates a large deviation from the SZP determined by the SP method (Fig. 6b). It has to be pointed out that outlying measurement data were not excluded.

#### 3.2.5 Effect of electrical conductance of solid sample

**Fig. 7:**
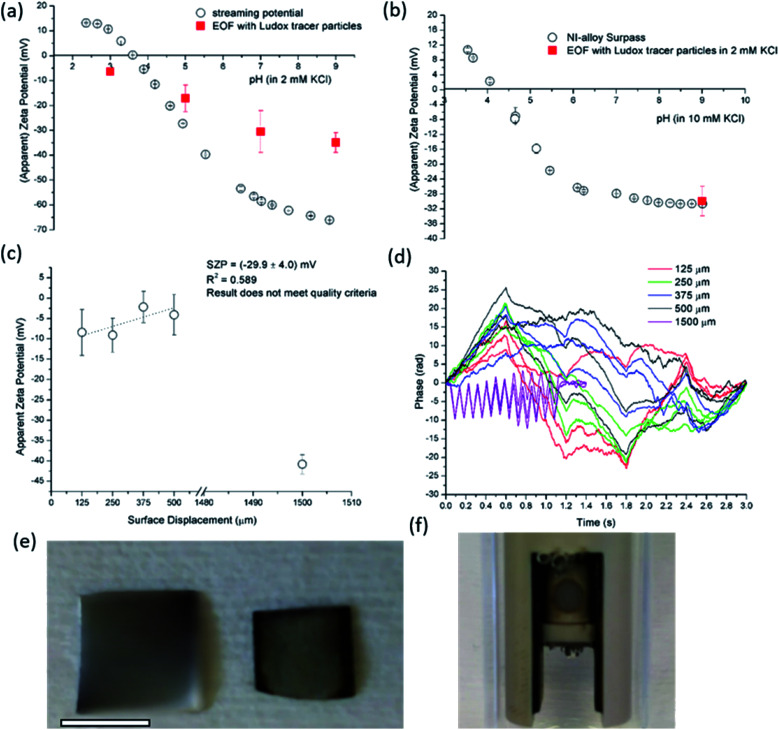
SP and EOF data using Ludox tracer particles for semiconductive silicon wafer (a). EOF measurement and SP data in all pH range for Ni-based conductive alloy (b). Apparent zeta potential *versus* surface displacements for Ni-based alloy at pH 9 and 2 mM ionic strength (c) with corresponding phase plots (d). The damage caused on the Ni-based alloy after EOF measurement in shown in ((e); right Ni-based alloy), where bar is 4 mm. Observation of possible electrochemical reaction with bubbles formation is presented in (f).

To elucidate the effect of the macroscopic solid material conductivity on the SZP analysis we selected a semi-conductive silicon wafer and a conductive Ni-based metal alloy as representative materials. Material conductance is an intrinsic material property and executes the most considerable effect on the SZP analyses. Additionally, metals can also account for electrochemical reactions with the aqueous solution and with the present solutes. Fig. 7a illustrates the SP data for the semi-conductive silicon wafer coated with a thin layer of silicon oxide. The SZP ranges from *ζ* = −66 mV at pH 9 to *ζ* = +13 mV at pH 2 with the IEP at pH 3.8. The latter is common for this type of material and in agreement with the IEP reported in the literature.^57^ Similarly to other SZP analyses, the SP results display minimum error bars with the relative uncertainly well below 10%. In spite of the similar trend of the SZP analyses by both the EOF and SP methods, the magnitude of the SZP at pH 9 is 50% lower (around *ζ* = −32 mV) for EOF in comparison to the SP results. Besides this, the relative measurement uncertainties with EOF are above 10% as shown by the large error bars. For example, at pH 7 the relative measurement uncertainty results in 28% at an absolute value of the SZP of *ζ* = −30.5 mV. This can be explained by the presence of the elevated conductivity that affects the integrity of the Ludox tracer particles, which again changed colour after the measurement. On the other hand, at pH 9 all three repetitions of the EOF measurements were of good quality and showed well-defined negative phase plots with minimum noise. Furthermore, as for other EOF experiments with the Ludox tracer particles an opposite sign of the SZP and the IEP were not obtained (Fig. 7a). The streaming current is not affected by material conductance and therefore allows for the SZP analysis of electrochemically inert metal surfaces,^58^ which is shown in Fig. 7b for a Ni-based alloy. Severe problems were observed for the conductive Ni-based metal foil using the EOF method that resulted in a low correlation coefficient of the linear regression fit (Fig. 7c) and erratic phase plots (Fig. 7d). Despite of the good correlation between the streaming current and the EOF results at pH 9 (Fig. 7b), a serious damage of the inert metal sample after concluding the EOF measurement was clearly revealed (Fig. 7e). Furthermore bubble formation on the tested surface was observed, which indicates electrochemical reactions triggered by the applied electric field (Fig. 7f). From this point of view it may be concluded that the streaming current measurement is applicable for the determination of the SZP of highly conductive samples thereby providing reliable and reproducible data, which contradicts the conclusion by Mateos *et al.*^14^ On the other hand, the EOF method indicated surface reactions (such as corrosion processes) at the metal sample and provided data of bad quality.

### 3.3 Repeatability, reproducibility and reliability of EOF and SP

#### 3.3.1 Repeatability

Table 1 represents the results of the SZP, the relative uncertainty and the linear correlation coefficient *R*^2^ for the RO membrane determined by both measuring methods in 2 mM KCl electrolyte solution at pH 9. Ludox tracer particles were used for the EOF measurement. To simulate the needs of the EOF method for a repetitive adjustment of the surface displacements, the distance between membrane surfaces was also re-adjusted for the SP measurement. A comparison of the repeated measurements using the same RO membrane clearly reveals the significant difference in the SZP obtained from EOF mapping and the SP method. The SP data shows a SZP repeatability with a relative uncertainty <10% and *R*^2^ > 0.95 while the SZP determined by the EOF method is more than 50% smaller in magnitude with a larger relative measurement uncertainty and linear regression fits that do not meet the quality criteria for an acceptable measurement.

**Table 1:** Repeatability of SZP for RO membrane determined by EOF and SP methods (2 mM KCl, pH 9)

EOF method			SP method		
SZP (mV)	Relative uncertainty (%)	*R* ^2^	SZP (mV)	Relative uncertainty (%)	*R* ^2^
−12.1 ± 4.5	37	0.716	−29.5 ± 0.4	1.3	0.999
−14.9 ± 2.5	17	0.881	−29.1 ± 0.3	1.2	0.999
−10.8 ± 3.6	33	0.806	−28.8 ± 0.4	1.2	0.999

#### 3.3.2 Reproducibility

**Fig. 8:**
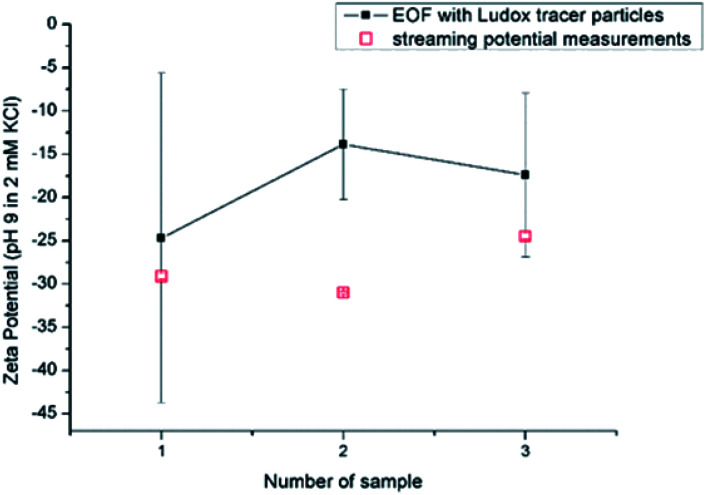
Reproducibility for RO membrane using EOF mapping with Ludox tracer particles and SP at pH 9 in 2 mM KCl.

The sample-to-sample reproducibility of the EOF and SP methods was tested for the RO membrane using three different samples of the same membrane sheet in 2 mM KCl solution at pH 9 (Fig. 8). The SP measurement reveals a reproducibility of ±11.9%, which is significantly worse than the repeatability for individual membrane samples. We interpret this scatter of the SZP by the heterogeneity of the membrane sample and of the equilibrium conditions at the membrane–water interface. Although the reproducibility obtained with the EOF method is in a similar range (±29.6%) the individual measurements show larger error bars and a SZP with a significantly smaller magnitude. The confidence in the SZP of the RO membrane is rather low for the results from the EOF approach. It is known that for materials that carry lower surface charge, the magnitude of the relative error is larger as, *e.g.*, for higher charged surfaces, with a large absolute SZP values such as glass or PVDF.^13,14^

#### 3.3.3 Reliability

The decision on the reliability of EOF results is generally based on existing measurements with the SP and streaming current method.^13–21^ Otherwise, the sometimes unpredictable scatter of EOF results makes it difficult to tell which of the results is feasible and which is subject to an artefact. For the study of macroscopic samples with challenging properties (porous, conductive), the EOF method shows larger measurement uncertainties, whereas SP and streaming current show low measurement errors regardless of the sample complexity. One of the primary goals of the SZP analysis is the determination of the IEP. When comparing both techniques, we observe problems to achieve the IEP by the EOF method regardless of the type of material, which is not the case for the SP measurement. Increased ionic strength lowers the reliability of data obtained with the EOF method, which limits its application for environmentally relevant conditions. Even in dilute solutions the EOF method fails to give reliable results at low pH due to the increased conductivity that causes changes also in the inorganic tracer particles' integrity. The analysis of oppositely charged solid surfaces and tracer particles revealed again the failure of the EOF method, which is caused by the unavoidable electrostatic attraction and requires the selection of appropriate tracer particles depending on the (unknown) charge of the solid surface. A damage of the sample was observed in the case of the metal alloy (highly conductive), which limits the use of the EOF method to non-conductive materials. Finally setting the zero position for the surface displacement correlation is problematic since it significantly affects the result of the EOF measurement.

## 4. Conclusions

In this report we compared in detail two different electrokinetic phenomena for the SZP determination, *i.e.* the electro-osmotic flow using tracer particles in a dip cell arrangement and the SP method. We took into account materials with different surface and bulk properties (roughness, porosity, electric conductivity) that are expected to influence the SZP analysis. For the validation of the EOF mapping method, we investigated the effects of the type of tracer particles, of the ionic strength, and of the applied voltage. The results revealed the unsuitability of the standard latex dispersion to be used as tracer particles at an electrolyte concentration exceeding 5 mM KCl, and at an applied voltage between palladium electrodes of 5 V. Two different silica-based tracer particles of different sizes were thus compared, and more reliable data for the EOF method were obtained with Ludox tracer particles. In general the indirect SZP analysis by the EOF method showed a large difference and significantly higher measurement uncertainty when compared to the SP method. Interestingly, for both negatively charged tracer particles neither the IEP nor a positive sign of the SZP were achieved for all solid samples studied. This was not the case for the SP method. We explain the failure to obtain a positive SZP with negatively charged tracer particles by a possible electrostatic attraction of particles to the solid surface. The effect of sample porosity was tested on using microfiltration membranes with different pore size, which lead to large measurement uncertainties for the EOF method, while minor error bars were obtained for the SP method. While the streaming current measurement allowed for a reasonable and reliable SZP for conductive samples, several obstacles were observed during EOF mapping that resulted even in the surface damage of a stainless metal sample. Measurement repeatability, reproducibility and reliability for selected samples are satisfactory when using the SP method, confidence in for the SZP obtained by EOF mapping is rather low. The EOF method disables the use of the environmentally relevant ionic strength due to tracer particle degradation.

The benefit of the EOF method is a smaller investment in the SZP accessory provided that a specific ELS instrument is already available but the disadvantages are as obvious. We observe a significant consumption of tracer particles, which likely degrade during the EOF measurement and require an exchange for every single measurement. Moreover, longer measurement times are required correlated with the high labour cost and longer measurement times. The manual adjustment of the surface displacements using the dip cell and the preparation of individual dispersions of tracer particles at each pH require a longer measurement time and the permanent attention of the operator. On the other hand, only a fraction of the measuring time and minimal user attention are required for the SP method.

In conclusion the reliability of SZP results obtained with the EOF method is recognized only by a comparison with the corresponding SP measurement.

## Conflicts of interest

There are no conflicts to declare.

## Supplementary Material

RA-010-C9RA10414C-s001

## References

[cit1] Hunter R. J. , Zeta potential in colloid science: Principles and applications, Academic Press, London, 1981

[cit2] Shaw D. J. , Introduction to colloid & surface chemistry, Butterworth-Heinemann, Oxford, 4th edn, 1992

[cit3] Elimelech M., Chen W. H., Waypa J. J. (1994). Desalination.

[cit4] Imbrogno A., Tiraferri A., Abbenante S., Weyand S., Schwaiger R., Luxbacher T., Schäfer A. I. (2018). J. Membr. Sci..

[cit5] Stana-Kleinschek K., Ribitsch V. (1998). Colloids Surf., A.

[cit6] Grancaric A. M., Tarbuk A., Pusic T. (2005). Color. Technol..

[cit7] Petrinić I., Bukšek H., Luxbacher T., Pušić T., Bischof S. (2018). J. Appl. Polym. Sci..

[cit8] Espanol M., Mestres G., Luxbacher T., Dory J. B., Ginebra M. P. (2016). ACS Appl. Mater. Interfaces.

[cit9] Lorenzetti M., Bernardini G., Luxbacher T., Santucci A., Kobe S., Novak S. (2015). Biomed. Mater..

[cit10] Pedimonte B. J., Moest T., Luxbacher T., Von Wilmowsky C., Fey T., Schlegel K. A., Greil P. (2014). Acta Biomater..

[cit11] Niepel M. S., Peschel D., Sisquella X., Planell J. A., Groth T. (2009). Biomaterials.

[cit12] Ribitsch V., Jorde C., Schurz J., Jacobasch H. J. (1988). Prog. Colloid Polym. Sci..

[cit13] Corbett J. C. W., Mcneil-watson F., Jack R. O., Howarth M. (2012). Colloids Surf., A.

[cit14] Mateos H., Valentini A., Robles E., Brooker A., Cioffi N., Palazzo G. (2019). Colloids Surf., A.

[cit15] Thomas T. E., Al Aani S., Oatley-Radcliffe D. L., Williams P. M., Hilal N. (2017). J. Membr. Sci..

[cit16] Cruz M. A. E., de Souza R. M., Dias L. G., Ramos A. P. (2017). Thin Solid Films.

[cit17] Al S., Wright C. J., Hilal N. (2018). Desalination.

[cit18] Shim Y., Lee H.-J., Lee S., Moon S.-H., Cho J. (2002). Environ. Sci. Technol..

[cit19] Vasconcelos J. M., Zen F., Stamatin S. N., Behan J. A., Colavita P. E. (2017). Surf. Interface Anal..

[cit20] Penfold N. J. W., Parnell A. J., Molina M., Verstraete P., Smets J., Armes S. P. (2017). Langmuir.

[cit21] Zhao C., Hu G., Hou D., Yu L., Zhao Y., Wang J., Cao A., Zhai Y. (2018). Sep. Purif. Technol..

[cit22] Hiratsuka K., Suzuki T., Dzieminska E., Ichiyanagi M. (2018). J. Fluid Sci. Technol..

[cit23] Kakihana Y., Cheng L., Fang L., Wang S., Jeon S., Saeki D. (2017). Colloids Surf., A.

[cit24] Rho H., Chon K., Cho J. (2018). Desalination.

[cit25] Suwarno S. R., Hanada S., Chong T. H., Goto S., Henmi M., Fane A. G. (2016). Desalination.

[cit26] Kukizaki M. (2009). Sep. Purif. Technol..

[cit27] Ishigami T., Amano K., Fujii A., Ohmukai Y., Kamio E., Maruyama T., Matsuyama H. (2012). Sep. Purif. Technol..

[cit28] Hongo-Hirasaki T., Komuro M., Ide S. (2010). Biotechnol. Prog..

[cit29] Hozumi A., Inagai H., Yokogawa Y., Kameyama T. (2003). Thin Solid Films.

[cit30] Han M. J., Baroña G. N. B., Jung B. (2011). Desalination.

[cit31] Park N., Lee S., Yoon Ro S., Hoon Kim Y., Cho J. (2007). Desalination.

[cit32] Jang J., Go W. (2008). Fibers Polym..

[cit33] Shon H. K., Kim S. H., Vigneswaran S., Ben Aim R., Lee S., Cho J. (2009). Desalination.

[cit34] Xu P., Drewes E., Kim T.-U., Bellona C., Amy G. (2006). J. Membr. Sci..

[cit35] Lee S., Cho J. (2004). Desalination.

[cit36] Burns N. L., Van Alstine J. M., Harris J. M. (1995). Langmuir.

[cit37] Jachimska B., Świątek S., Loch J. I., Lewiński K., Luxbacher T. (2018). Bioelectrochemistry.

[cit38] Vandenbossche M., Dorst J., Amberg M., Schütz U., Rupper P., Heuberger M., Hegemann D. (2018). Polym. Degrad. Stab..

[cit39] Croisier F., Sibret P., Dupont-Gillain C. C., Genet M. J., Detrembleur C., Jerome C. (2015). J. Mater. Chem. B.

[cit40] Croisier F., Atanasova G., Poumay Y., Jérôme C. (2014). Adv. Healthcare Mater..

[cit41] Glaser T. K., Plohl O., Vesel A., Ajdnik U., Ulrih N. P., Hrnčič M. K., Bren U., Zemljič L. F. (2019). Materials.

[cit42] Plohl O., Ajdnik U., Gyergyek S., Ban I., Vesel A., Glaser T. K., Zemljič L. F. (2019). J. Environ. Chem. Eng..

[cit43] Plohl O., Finšgar M., Gyergyek S., Ajdnik U., Ban I., Fras Zemljič L. (2019). Nanomaterials.

[cit44] Uzgiris E. E. (1981). Prog. Surf. Sci..

[cit45] Bauman M., Košak A., Lobnik A., Petrinić I., Luxbacher T. (2013). Colloids Surf., A.

[cit46] Ashraf K. M., Giri D., Wynne K. J., Higgins D. A., Collinson M. M. (2016). Langmuir.

[cit47] Wang C., Zolotarskaya O., Ashraf K. M., Wen X., Ohman D. E., Wynne K. J. (2019). ACS Appl. Mater. Interfaces.

[cit48] Zimmermann R., Freudenberg U., Schweiß R., Küttner D., Werner C. (2010). Curr. Opin. Colloid Interface Sci..

[cit49] Owusu-Agyeman I., Jeihanipour A., Luxbacher T., Schäfer A. I. (2017). J. Membr. Sci..

[cit50] Tkavc T., Petrinič I., Luxbacher T., Vesel A., Ristić T., Zemljič L. F. (2014). Int. J. Adhes. Adhes..

[cit51] Ristić T., Persin Z., Kralj Kuncic M., Kosalec I., Zemljic L. F. (2019). Text. Res. J..

[cit52] Fras-Zemljič L., Kosalec I., Munda M., Strnad S., Kolar M., Bračič M., Šauperl O. (2015). Cellulose.

[cit53] Ajdnik U., Zemljič L. F., Bračič M., Maver U., Plohl O., Rebol J. (2019). Materials.

[cit54] Zemljič L. F., Plohl O., Vesel A., Luxbacher T., Potrč S. (2020). Int. J. Mol. Sci..

[cit55] Yaroshchuk A., Luxbacher T. (2010). Langmuir.

[cit56] Childress A. E., Deshmukh S. S. (1998). Desalination.

[cit57] Schwarz S., Eichhorn K. J., Wischerhoff E., Laschewsky A. (1999). Colloids Surf., A.

[cit58] Martin Cabanas B., Lützenkirchen J., Leclercq S., Barboux P., Lefèvre G. (2012). J. Nucl. Mater..

