# Supplementary material for: Applicability of electro-osmotic flow for the analysis of the surface zeta potential
Source: RSC Adv. 2020 Feb 13;10(12):6777–89. doi: 10.1039/c9ra10414c (PMC9049723; doi:10.1039/c9ra10414c)
Supplement: RA-010-C9RA10414C-s001 [file RA-010-C9RA10414C-s001.pdf]

Electronic Supplementary Material (ESI) for RSC Advances.
This journal is © The Royal Society of Chemistry 2020

**Supporting Information for:**

# Applicability of electro-osmotic flow for the analysis of the surface zeta potential

Olivija Plohl[1,*], Lidija Fras Zemljič[1], Sanja Potrč[1,2], Thomas Luxbacher[3]

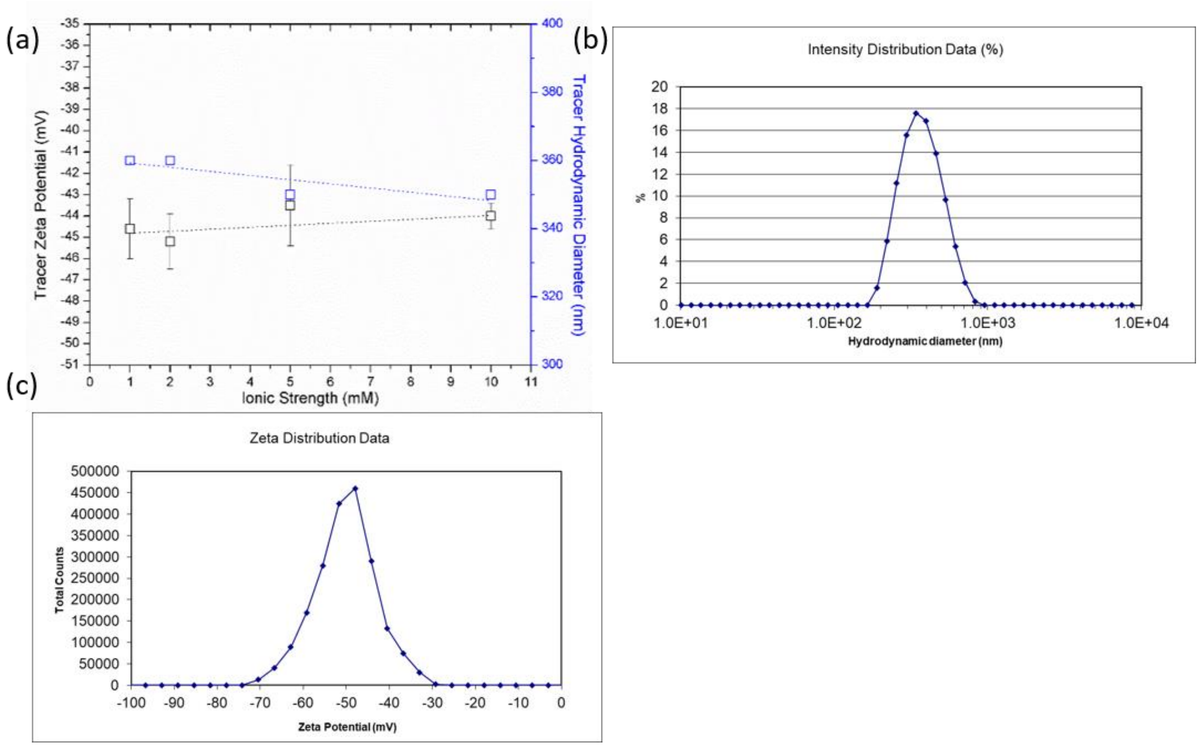


Figure SI1: (a) ZP and hydrodynamic diameter of Latex tracer particles as a function of ionic strength. (b) Example of latex tracer particles size distribution and (c) zeta potential graph in 2 mM KCl solution and pH 9.

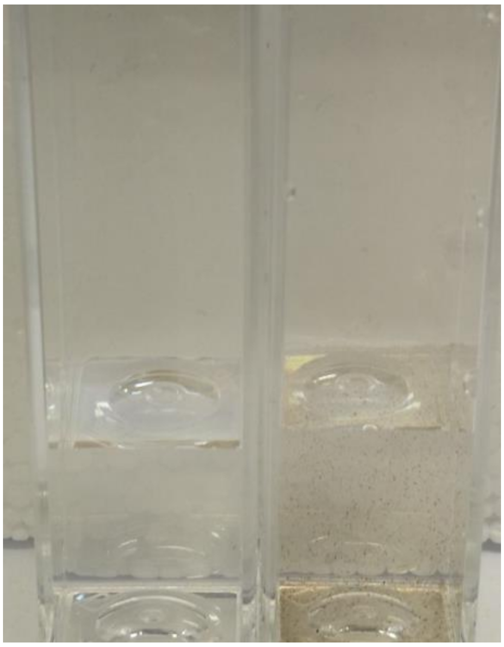



Figure SI2: Image of Latex tracer particle dispersion before and after measurement of the PEEK sample holder at 5 mM KCl in pH 8-9.

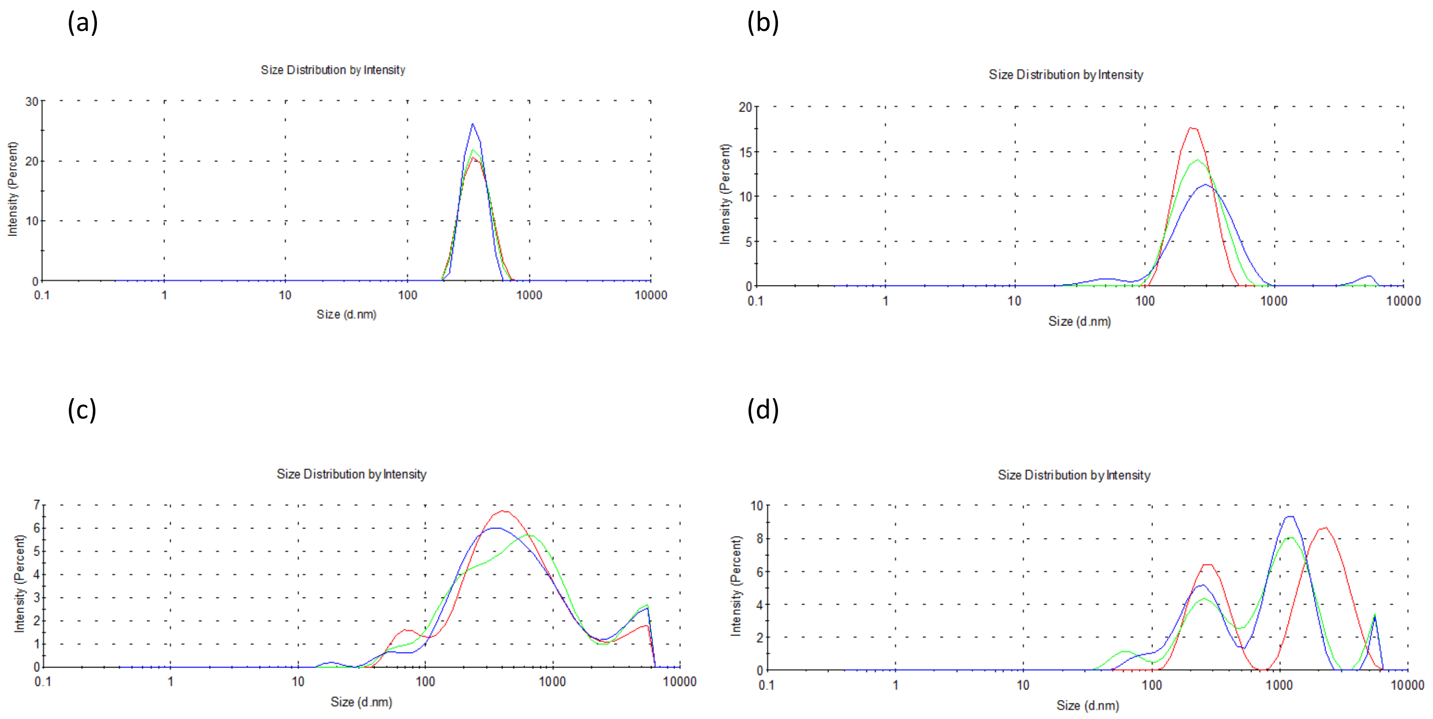


Figure SI3: Tracer latex particles in the 5 mM KCl before measurement (a); the same tracer particles after the measurement at 5 V (b), 10 V (c) and 20 V (d).

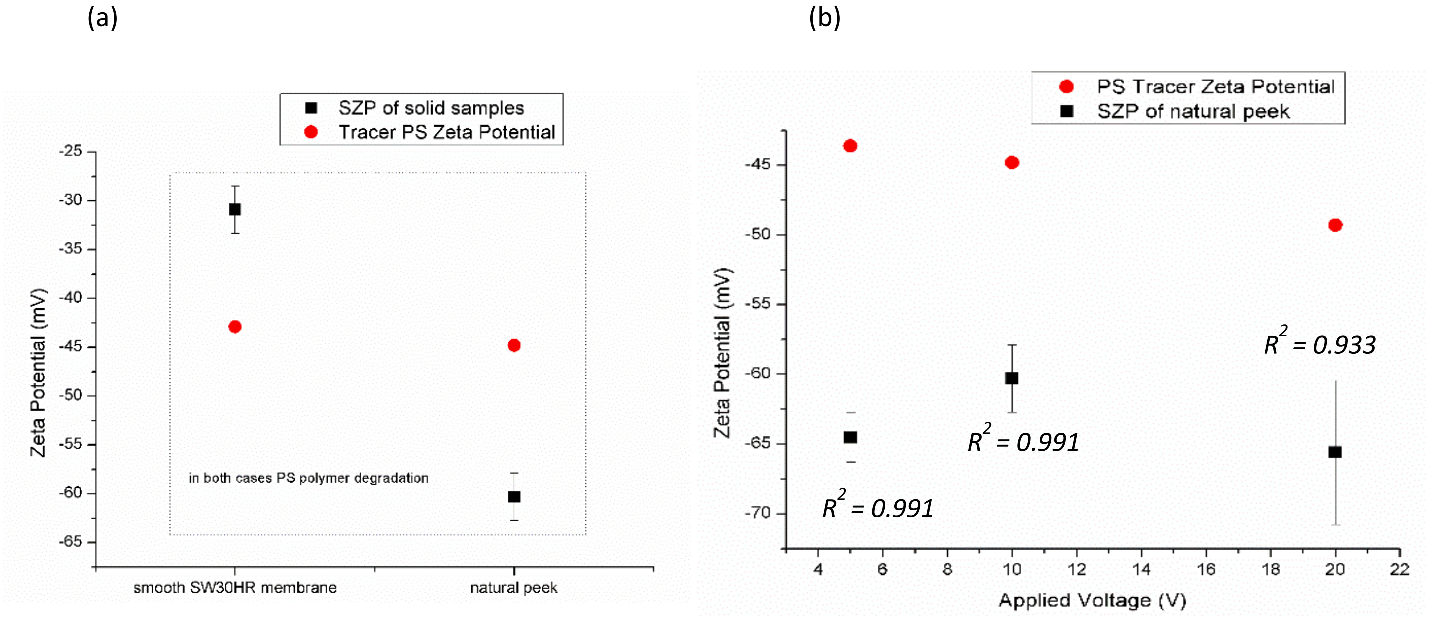


Figure SI4: Effect of sample onto Latex tracer particle stability in 5 mM KCl (a) and effect of the manually applied voltage onto extend of the polymer tracer particles degradation (b).

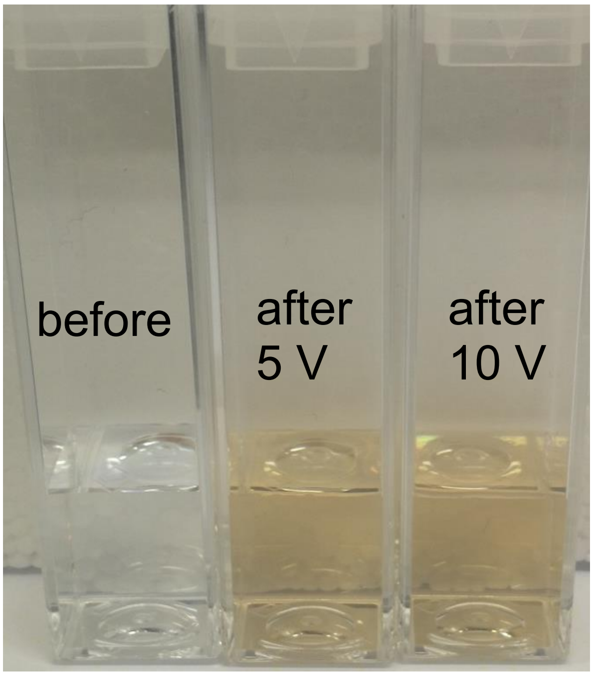

Figure SI5: Latex tracer particle dispersion before and after measurement manually applied voltage 5 V and 10 V in 5 mM KCl.
